# Lithium in Cancer Therapy: Friend or Foe?

**DOI:** 10.3390/cancers15041095

**Published:** 2023-02-08

**Authors:** Chunhao Yang, Bo Zhu, Mingjie Zhan, Zi-Chun Hua

**Affiliations:** 1 School of Biopharmacy, China Pharmaceutical University, Nanjing 211198, China; 2 The State Key Laboratory of Pharmaceutical Biotechnology, School of Life Sciences, Nanjing University, Nanjing 210023, China

**Keywords:** lithium, cancer therapy, cell death, tumor immune microenvironment

## Abstract

**Simple Summary:**

Lithium has served as a mental stabilizer since the 20th century. Several recent studies have demonstrated the antitumor effects of lithium. Lithium can also mitigate chemotherapy side effects, such as cachexia. This review summarizes lithium’s role in tumor development, and discusses the relevant underlying mechanisms.

**Abstract:**

Lithium, a trace element important for fetal health and development, is considered a metal drug with a well-established clinical regime, economical production process, and a mature storage system. Several studies have shown that lithium affects tumor development by regulating inositol monophosphate (IMPase) and glycogen synthase kinase-3 (GSK-3). Lithium can also promote proliferation and programmed cell death (PCD) in tumor cells through a number of new targets, such as the nuclear receptor NR4A1 and Hedgehog-Gli. Lithium may increase cancer treatment efficacy while reducing side effects, suggesting that it can be used as an adjunctive therapy. In this review, we summarize the effects of lithium on tumor progression and discuss the underlying mechanisms. Additionally, we discuss lithium’s limitations in antitumor clinical applications, including its narrow therapeutic window and potential pro-cancer effects on the tumor immune system.

## 1. Introduction

Traditional cancer treatment approaches, including chemotherapy, radiotherapy, and surgical therapy, meet the problems of tumor tissue remnant, acute side effects, narrow application scope, and drug resistance [1,2]. Compared with traditional therapy, metal ion treatment can kill tumor cells with fewer side effects and less drug resistance [3]. Metal ions are not immunogenic, but are involved in regulating the tumor immune microenvironment [3,4]. These advantages suggest that metal ions may play a greater role in tumor therapy in the future.

Lithium is a classical drug with a mature treatment program and monitoring system, and it is stable in most conditions and suitable for transportation and storage [5]. Lithium is more safe and reliable under limited dosage compared with platinum [6,7]. Moreover, lithium salts do not need the ionophore compound to form a complex, thus it is cheap enough for production and transportation [8].

Since the 20th century, lithium treatment has been considered a gold standard for bipolar disorder (BD) and applied to psychosis and mental health conditions [9,10]. According to the “inositol depletion hypothesis”, lithium directly inhibits inositol mono-phosphatase (IMPase) and prevents inositol monophosphates from dephosphorylating to inositol [11]. Lack of inositol reduces the production of phosphatidylinositol and down-regulates phosphatidylinositol 4,5-bisphosphonate. Therefore, less phosphatidylinositol 4,5-bisphosphonate is broken down by phospholipase C to form a secondary messenger, myo-inositol-1,4,5-triphosphate (IP_3_), which blocks the signal transmission of aberrant neurotransmitters in pathological regions [12,13,14]. According to another widely-accepted “glycogen synthase kinase-3β (GSK3β) inhibition hypothesis”, aberrant expression of GSK3β activates the phosphatidylinositol 3-kinase/protein kinase B (PI3K/Akt) pathway and blocks the adjustment of insulin [15]. While lithium is considered a GSK3β inhibitor to recover insulin signals, it enhances glucose metabolism and alleviates the process of neuro-progression [15,16].

Overexpression of GSK3β appears in various types of tumors, suggesting that lithium may have potential in cancer therapy [17]. In the 1980s, lithium was first discovered to prevent radio-induced leukemia [18]. Since then, lithium has gained increasing attention for its potential antitumor properties. Currently, lithium has been used in clinical trials for the treatment of leukopenia and the ablation of tumors [19,20]. Additionally, a meta-analysis showed that lithium exposure reduced morbidity and mortality in some cancer patients [21,22,23]. The aim of this review is to summarize the tumor-biological effects of lithium and to explore the potential applications and challenges of lithium in cancer treatment.

## 2. Direct Protein Targets of Lithium

**Figure 1:**
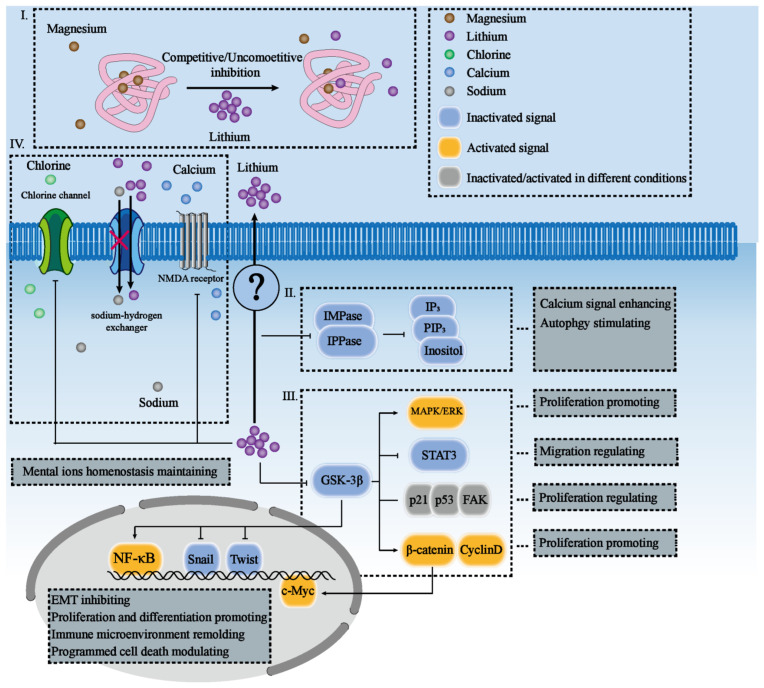
Pathways that are directly regulated by lithium. (I) High-concentration lithium ions take away metal binding sites, causing magnesium-dependent enzymes to lose their functions. (II) Inhibition of IMPase by lithium promotes calcium signaling and autophagy, but suppresses proliferation in cells. (III) Inhibition of GSK-3β by lithium activates the MAPK/ERK and β-catenin pathways, but inactivates STAT3 signaling, thus affecting the survival, proliferation, and migration of cells. (IV) Lithium affects the intracellular homeostasis of other ions, such as calcium, sodium, and chlorine.

As an essential mineral nutrient, lithium is mainly supplemented through dietary intake and presents as free ions inside body fluid [24]. Normally, for an adult receiving 1.78 mg lithium per day for several weeks, the concentration of lithium in the serum will be stable at around 2.6 μmol/L [24]. Evidence linked low lithium intakes with altered behavior and aggressiveness in humans [24]. While it is still unclear how lithium exhibits biological functions at nutritional levels, some direct targets of lithium are found at the pharmacological level (Figure 1).

The strong polarization property of lithium ions makes metalation happen in the metal binding site of other types of metal ions, including potassium, calcium, sodium, and magnesium in various enzymes [25]. Especially, lithium prefers to inhibit magnesium-dependent enzymes because of the similar radius of these two ions [25,26]. Importantly, the three known direct targets of lithium contain magnesium binding sites with different structures, including IMPase, GSK-3, and magnesium-dependent phosphate monoesterases.

Lithium inhibits GSK3β activity in different ways. Firstly, lithium ions compete with the magnesium binding site directly to inhibit the catalysis activity of GSK3β [27]. Secondly, lithium is rooted in the active site groove of GSK3β and closes the active site entrance with a shorter distance between three pairs of “Pocket mouth” residues (21 and 7; 217 and 5; 260 and 65) [28]. Thirdly, lithium alters the affinity of GSK3β with its chaperones, and this then activates the pathways that are suppressed by GSK3β [28]. Fourthly, lithium may inactivate GSK3β indirectly via the PI3K/Akt pathway activation. Lithium activates PI3K, which phosphorylates the Thr308 of Akt, then mediates the phosphorylation of Ser9 in GSK3β [29,30,31]. Phosphorylated Ser9 can occupy the priming phosphorylation site, and block the substrate activity to inactivate GSK3β [30]. As a widely-known oncogene targeted by lithium, GSK-3 also affects the activities of various protein kinases, cytoskeletal proteins, and transcription factors, thus promoting cell metabolism and proliferation [25].

Unlike GSK3, lithium directly inhibits IMPase and other magnesium-dependent phospho-monoesterases in an uncompetitive manner [32]. In 1995, John et al. found that all of these lithium-inhibited enzymes, such as IMPase (Ki = 0.8 mM), inositol polyphosphate-1-phosphatase (Ki = 0.3 mM), and fructose 1,6-biphosphatase (Ki = 0.3–0.8 mM), shared a common sequence motif (Asp-Pro-(Ile or Leu)-Asp(Gly or Ser)-(Thr or Ser)) responsible for metal binding and catalysis [33]. Further research in crystal structure mentioned a three-metal-assisted mobile loop in IMPase that helps initiate the catalysis cycle when the second and third metal ions are transiently localized close, to create the water nucleophile [34]. Lithium is small enough to replace magnesium in a three-metal-assisted mobile loop. However, the mobile loop with lithium cannot hydrate the phosphate group and transfer it to the substrate, because lithium lacks charges and fails to form the water-based nucleophile [34]. Emerging evidence supported the three-metal hypothesis and illustrated a precise IMPase–2Mg^2+^–Li^+^ complex model for IMPase inhibition [26]. It also mentioned that lithium could further prevent the protonation of the inositol group after phosphoester hydrolysis, trap inositol and monophosphate in the active site [26]. Importantly, inhibition of IMPase and other magnesium-dependent phosphomonoesterases decreases intracellular inositol signal transduction and affects cell proliferation [13].

Lithium also affects the intracellular homeostasis of sodium and calcium. Lithium reduces intracellular sodium levels via a sodium–lithium exchange system, labeled as a sodium–lithium counter-exchange pump [35]. Emerging evidence indicates that lithium reduced sodium levels and enhanced the intracellular pH value [36]. Moreover, the sodium–hydrogen exchanger (NHE) inhibitor, ethyl-isopropyl amiloride, completely inhibited the intracellular alkalinization induced by lithium, suggesting that the sodium–lithium counter-exchange pump was one of the NHE isoforms [36,37]. Lithium can also reduce the level of intracellular calcium (describing in Section 3.6).

Moreover, lithium can inhibit the volume-activated chloride channel and prevent the influx of chloride. By preventing the influx of chloride, lithium prevents the loss of cell regulatory volume under hypotonic conditions [38]. Similar effects of lithium and other GSK3β inhibitors on the volume-activated chloride channel suggest the involvement of GSK3β in this process [38].

## 3. Anti-Cancer Effects and Underlying Mechanisms of Lithium

### 3.1. Lithium Regulates the Process of Programmed Cell Death (PCD)

#### 3.1.1. Apoptosis

Lithium induces PCD, such as apoptosis and autophagy, rather than necrosis [39]. It induces apoptosis with DNA fragmentation and phosphatidylserine eversion in various types of cancer cells, including colorectal cancer, melanoma, pancreatic cancer, thyroid cancer, and leukemia [40,41,42,43,44].

Apoptosis can be triggered through the extrinsic and intrinsic pathways. The extrinsic apoptosis is often triggered by the stimulation of the death receptors (DRs) with their ligands, such as tumor necrosis factor (TNF)-related apoptosis-inducing ligand (TRAIL) and Fas ligand (FasL) [45]. Lithium was reported to enhance the apoptotic effects of TRAIL by up-regulating the expression of DR4 and DR5 in lung cancer cells [46].

Different from the extrinsic pathway, intrinsic apoptosis is activated by the release of apoptogenic factors, such as cytochrome c, an apoptosis-inducing factor (AIF), and regulated by the B-cell lymphoma 2 (Bcl-2) protein family [45]. Lithium can downregulate the anti-apoptotic protein Bcl-2 and up-regulate the pro-apoptotic protein Bax [40,47]. Furthermore, the metabolic reprogramming marker NR4A1 was reported to shift Bcl-2 to a cytotoxic protein, as it is located in the cytoplasm [48]. Lithium increased NR4A1 expression in leukemia cells [43], suggesting that lithium may promote apoptosis via NR4A1 signaling regulation. Therefore, the Bcl-2 family plays an essential role in the mechanisms of lithium cytotoxicity. Lithium was also demonstrated to reinforce the efficiency of other apoptosis inducers, such as mitomycin c, HDAC inhibitors, arsenic trioxide, and sorafenib [47,49,50,51].

It is worth mentioning that Antoni Camins et al. demonstrated that lithium triggered apoptosis without caspase3 cleavage and AIF change in neuroblastoma cells [52], suggesting that various mechanisms may be involved in cell death induced by lithium.

#### 3.1.2. Autophagy

Lithium induces autophagy with the appearance of autophagosomes and autolysosomes in several cancers, including melanoma, hepatoma, cervical cancer, and renal carcinoma [41,53,54,55,56]. The bio-markers LAMP1 and LC3II, located on the surface of mature and integral autophagosomes, are up-regulated after lithium treatment [56,57,58]. However, it is still unclear how lithium triggers autophagy. One of the explanations is that lithium induces low-concentration IP_3_ and triggers autophagy via an inhibition of IMPase [56]. While the IP_3_ pool recruits RAB7A to bind the homotypic fusion and protein-sorting complex, the complex locates on the lysosome and then mediates the merging of the autophagosome and lysosome [59]. Another possible explanation is that lithium promotes clathrin-independent endocytosis, modulates endosomal recycling, and induces autophagy [60].

The relationship between autophagy and cancer development is complex. Apart from providing metabolic materials and energy to tumor cells, autophagy degrades oncogenes and misfolded proteins, thus reducing tumor occurrence [61]. The collapse of autolysosomes by lithium may also cause fatal damage to cancer cells [62].

#### 3.1.3. Necroptosis

Bilir et al. found that prostatic cancer cells showed necrotic structures after 1–10 mM lithium treatment [63]. Besides, Wang et al. found that the death of schwannoma cells triggered by 20 mM lithium could be reversed by the necroptosis inhibitor Nec-1 [64]. Iran et al. found that lithium can inhibit cell necrosis caused by mitomycin c in breast cancer cells [49]. They also found that the cleaved poly (ADP-ribose) polymerase, a classical apoptosis marker, was up-regulated with lithium and mitomycin c treatment, suggesting that lithium may transfer necrosis to apoptosis [49]. Thereby, lithium tends to lead to necroptosis rather than necrosis.

### 3.2. Lithium Affects Cell Proliferation

**Figure 2:**
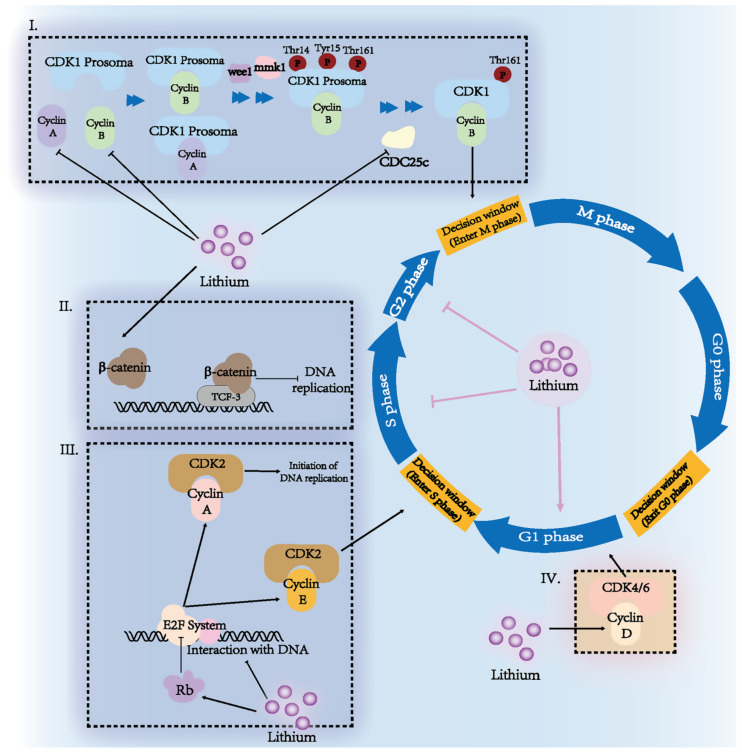
Effects and underlying mechanisms of lithium on the cell cycle in tumor cells. Lithium regulates cell proliferation mainly through cell cycle arresting and DNA replication blocking. A higher portion of G2/M phase and S phase cells has been observed in different tumor cells. (I) Lithium inactivates CDK1 by preventing CDC25c from hydrolyzing phosphorylated fragments, then prevents cells from entering the M phase. (II) For DNA replication, the S phase-specific interaction between TCF-3 and β-catenin shows an anti-proliferation effect, which might explain the S phase arresting by lithium. (III) Lithium disrupts the interaction between DNA and E2F systems. Lithium also improves the level of Rb to influence Cyclin A and Cyclin B indirectly. (IV) There is also a promotion effect of lithium to up-regulate the level of Cyclin D and then help tumor cells re-enter a new cell cycle.

Lithium treatment inhibits the growth of most cancer cells at high concentrations (more than 10 mM). Lithium’s function as a tumor growth inhibitor can be understood in the context of cell cycle modulation and DNA metabolism. The inhibitory effects of lithium on cell proliferation are shown in Figure 2.

#### 3.2.1. Lithium Arrests Cells in the S and G2/M Phases

Lithium is believed to arrest cells in the S phase and G2/M phase in different temporal features. One study showed that the S phase and G2/M phase increased simultaneously in leukemia cells [44] and glioblastoma cells [65] after lithium stimulation for 24 h. According to other studies, esophageal cancer cells and medulloblastoma cells were arrested in the S phase after lithium stimulation at 6 h, but returned to their basal levels after 24 h. Simultaneously, esophageal cells in the G2/M phase increased after treatment for 24 h [66,67]. Therefore, specific characteristics were exhibited in different types of cancers for the lithium effects on cell cycle.

Mechanistically, lithium indirectly influences the cell cycle checkpoint via an inhibition of GSK3β [52,68,69,70]. Inhibition of GSK3β activates the Wnt pathway, reduces the degradation of host transcription factors, such as c-Myc and Cyclin D1, and then regulates the cell cycle systematically [71].

Firstly, lithium suppressed E2F-dependent transcriptional network; the primary system encodes critical proteins in the cell cycle and DNA replication. Lithium destructed the bond between E2F1 and DNA, thus down-regulated Cyclin A and Cyclin E, and inhibited the initiation of the S phase [72]. Evidence also showed that lithium-tolerant leukemia cells expressed lower retinoblastoma protein (Rb) than their parental controls [73]. Lithium also promoted Rb expression to inhibit the E2F system [73].

Secondly, lithium also regulates CDK1 activation through an E2F-independent pathway. In this part, activation of CDK1 requires three steps: Cyclin A or Cyclin B combination, Weel/mik1 phosphorylation, and excision of Thr14/Tyr15-phosphorylated fragments by cell division cycle 25c (CDC25c) [74]. Lithium down-regulated CDC25c in adenocarcinoma cells [72]. Lithium also significantly increased Tyr15-phosphorylated CDK1 [52]. Therefore, lithium may suppress the maturity of CDK1 via downregulating CDC25c. Accumulation of inactivated CDK1 inhibits the formation of the M phase checkpoint, the CDK1–Cyclin B complex after DNA replication, and prevented cell division.

Thirdly, lithium inactivates the Hh–Gli pathway to prevent the expression of several cell cycle regulators. Abnormal activation of Hh–Gli up-regulates the expression of Cyclin D1, Cyclin D2, E2F1, and N-Myc, and promotes proliferation in multiple cancers, such as head and neck squamous-cell carcinoma, pancreatic ductal adenocarcinoma, and medulloblastoma [42,69,70]. Lithium lowered Gli activity in various cancer cells [70]. In oral squamous-cell carcinoma, lithium stimulated the Ser9 phosphorylation of GSK3β, and led to Gli1 degradation [70]. Lithium also promoted the formation of an inactive Gli3 in head and neck squamous-cell carcinoma [69]. Thus, Hh–Gli might be an essential target for the anti-proliferative effect of lithium.

However, lithium also increased the level of Cyclin D and its cooperator CDK4, promoting the re-entry of cells into the cell cycle in neuroblastoma [52], which indicated a broader network of lithium for cell cycle regulation.

#### 3.2.2. Lithium Inhibits DNA Replication and Reparation

Lithium inhibits DNA replication in several cancer types, mainly via the activation of the Wnt/β-catenin pathway. [68,72,75]. β-catenin is an effector of the Wnt pathway, activated by the lithium-stimulated phosphorylation of GSK3β [76]. Typically, β-catenin is a pro-proliferation protein, interacting with TCF-4 for its function [77]. Emerging evidence showed a new regulation model of β-catenin to change its cooperator to TCF-3-specific in the S phase, then exhibit an anti-proliferation effect [78]. Thus, lithium promotes cancer cells to re-enter the cell cycle, but simultaneously inhibits DNA replication and blocks them in the S phase. Moreover, this finding may partly explain the multi-functions of lithium on tumor cell proliferation.

Lithium also inhibits the intrinsic DNA repair capacity of cells. The DNA repair system prevents cells from double-strand, break-induced cell death, which may weaken the antitumor effect of ionizing radiation [79]. Lithium significantly reduced the protein level of p57, a protector of DNA from extracellular damage [80]. Lithium also inhibited DNA reparation in breast carcinoma, by down-regulating MRE11 levels [81,82]. As a consequence, high-concentration lithium treatment enhanced radiotherapy sensitivity significantly [80,81,83]. These findings suggest that lithium may be promising for radiotherapy.

### 3.3. Lithium Prevents Tumor Metastasis

#### 3.3.1. Lithium Reverses Epithelial–Mesenchymal Transition (EMT)

Lithium was found to reverse the EMT process with the cytoskeleton retracted function in tumor cells [84]. Rosa et al. further found that long-term treatment with lithium down-regulated mesenchymal cell markers Vimentin and N-cadherin, and up-regulated epithelial cell marker E-cadherin in EMT-induced colon cancer cells [85]. Lithium acted as a GSK3β inhibitor to inactive NF-κB, then prevented the expression of two NF-κB targets: Snail and Twist [84,85]. While Snail and Twist initiate tumor cells to transfer their properties close to mesenchymal cells, they can diffuse better around the body [86]. Therefore, lithium triggers a functional cross-regulation between Wnt and NF-κB pathways, leads to the reduction of Snail and Twist, then inhibits metastasis by reversing EMT.

#### 3.3.2. Lithium Inhibits Blood Vessel Development and Lymphangiogenesis

Abnormal blood vessels in the tumor tissue are considered a key factor contributing to metastasis. In the chicken chorioallantoic membrane, lithium reduced vessel branch points in a dosage-dependent manner [87]. Lithium also inhibited blood vessel development in rat entorhinal cortex cells [88]. Apart from down-regulating blood vessel counts, lithium also decreased the expression of CD31/LYVE1 in the lung and lymph nodes by decreasing the level of transforming growth-factor-induced protein [89]. Collectively, these studies suggest that lithium may inhibit tumor metastasis by inhibiting blood vessel development and lymphangiogenesis.

### 3.4. Lithium Modulates Intracellular Redox Balance

Lithium reduced SOD/CAT ratios and ROS production by remitting mitochondria dysfunction in BD therapy [69,70,71,90,91,92]. In contrast, lithium promoted ROS generation and lipid peroxidation in the kidney and liver at toxicity dosages in rats [90,93]. Additionally, high-concentration lithium (over 10 mM) stimulates the production of ROS in a dosage-dependent manner in colorectal cancer cells [16]. Based on these findings, high lithium concentrations are likely to increase oxidative stress, while low lithium concentrations tend to reduce oxidative stress.

### 3.5. Lithium Remolds Energy Metabolism

In 2001, Repetto et al. reported that 24 μM lithium inhibited hexosaminidase activity, while 0.24 mM lithium inhibited hexosaminidase release in neuro gliomas [94], suggesting that lithium has a significant impact on intracellular metabolism before it influences cell proliferation. Furthermore, Beitner et al. found that lithium improved the separation of hexokinase from mitochondria in melanoma cells, inactivated hexokinase, and prevented glycolysis for ATP production [95].

### 3.6. Lithium Down-Regulated Intracellular Calcium

Dyshomeostasis of calcium influences cell cycles and PCDs, and links calcium homeostasis with tumor development [96]. Lithium could down-regulate intracellular calcium levels mainly through an inhibition of calcium influx, as well as the degradation of extruding calcium channels. Lithium phosphorylated Tyr1742 of the NR2B subunit of the NMDA receptor, a calcium channel protein, and blocked calcium influx [97]. It was also reported that lithium inactivated calpain, because of low intracellular calcium levels [98,99]. Calpain is another calcium regulator that mediates the cleavage of the sodium/calcium pump NCX3 to prevent the extruding of calcium, and lithium prevented the degradation of NCX3 to down-regulate intracellular calcium [100,101]. Additionally, reducing the IP_3_ signal by lithium inhibits the release of calcium from the endoplasmic reticulum [101,102]. The way in which lithium plays a role in tumor therapy by affecting calcium signaling deserves further study.

### 3.7. Lithium Nourishes the Muscle

Cachexia is a common tumor complication. Lithium promoted myotube formation, increased muscle regulatory factors, and muscle-specific protein expression, suggesting it may improve cachexia in tumor treatment [103]. The nourishing efficiency of lithium not only attenuates cell death, but also promotes the differentiation of muscle. Since 2013, lithium was found to prevent muscle cells from damage by oculopharyngeal muscular dystrophy [104]. While the inhibition of GSK3β activated the Wnt pathway, lithium up-regulated the Wnt downstream target proteins, such as Bcl-2 and CREB to survive the muscle cells from the PABPN1, mutant-caused damage [104]. Another study found that GSK3β plays a central role in regulating myogenic differentiation and contributing to muscle atrophy [105]. Lithium, as the GSK3β inhibitor, activated the insulin signal to decrease the expression of atrogin-1 and MuRF1, two muscle-specific ubiquitin E3 ligases [105]. It was found that lithium could ameliorate myosin-heavy chain degradation, myotube wasting, and macrophage-induced inflammation, and reverse the cachexia in colorectal tumor-bearing mice [103]. However, the cachexia-inhibitory effects of lithium need further confirmation in more cancer types.

## 4. Applications of Lithium to Cancer Therapy

### 4.1. Effects of Lithium on Tumor Development and Incidence

Lithium prevents tumor development in specific cancers. Lithium treatment suppressed the formation of abdominal aortic aneurysms in a rat model [106]. Lithium also significantly prolonged the survival of mice with leukemia or melanoma [107,108]. A population-based study reported that BD patients with lithium treatment had a lower melanoma-associated mortality rate [23]. Another clinical study that included 40 patients with AML showed that lithium carbonate improved remission rates and survival time [109]. Lithium, a GSK3β inhibitor, can cooperate with retinoic acid (RA) for AML treatment by inducing the differentiation of AML stem cells [110]. Boisvert et al. reported that a 65-year-old male patient with small-cell lung cancer had tumor regression without recurrence after 12 years of high-dosage lithium treatment [21]. For small-cell lung cancer, a lithium carbonate-treated group also had a higher objective response rate and more extended survival period than the control group [111]. For inoperable pancreatic cancer, patients with lithium treatment at a high dosage exhibited a longer survival time than those with a low dosage [112]. Collectively, much evidence shows lithium prevents the development of different cancers, including abdominal aortic aneurysms, leukemia, melanoma, lung cancer, and pancreatic cancer.

In 2015, the European Medicines Agency reported that long-term lithium treatment might cause microcysts, oncocytomas, and collecting duct renal carcinomas [113]. Nevertheless, Ambrosiani et al. reviewed the Cagliari clinical database for lithium-treated cases from 1980 to 2013, and determined that lithium treatment was not associated with thyroid and renal cancers [114]. Simon et al. pointed out that long-term lithium treatment might induce abnormal hyperparathyroidism and lead to adenomatous transformation in the parathyroid gland [115]. In contrast, lower cancer risk was reported in BD patients who received long-term lithium treatment [22]. Hence, it is still unclear whether lithium participates in thyroid gland and urinary system disorders, and induced related cancers. Clinical research about cancer therapy using lithium is summarized in Table 1.

### 4.2. Adjuvant Effect of Lithium with Other Anti-Cancer Drugs

Although lithium alone at pharmacological concentrations has antitumor effects in some specific mouse models, a combination with other drugs may allow lithium to be used more widely in clinical applications. More rational, hypothesis-driven manners of lithium combinations were proposed based on the better-defined action of lithium.

For example, lithium was confirmed to have cytotoxic effects via DRs and FasL-mediated apoptosis, it enhanced the cytotoxicity of extrinsic apoptosis inducers, TRAIL and temozolomide [46,129]. For apoptosis-incompetent cells, lithium consistently illustrated chemosensitizing activity combined with 5-fluorouracil [62]. Although the nature of the cell death induced by lithium and 5-fluorouracil has not been accurately defined, this combination significantly increased the survival rate in colorectal tumor-bearing mice [62]. As the GSK3β inhibitor, lithium phosphorylated ERK1/2 to synergize with RA, helping induce the differentiation of RA-resistant AML cells [107]. Considering the efficiency of DNA metabolism, lithium was used to enhance photon therapy efficacy for colorectal cancer [83]. While BRAF-mutated melanoma acquires drug resistance when HuR is overexpressed [108], lithium was demonstrated to suppress the proliferation of HuR-expressed cells and alleviate drug resistance [108].

Lithium further blocks the side effects of several first-line antitumor drugs. For example, lithium remitted the peripheral neuropathy induced by paclitaxel via its neuron-protected function [132]. Eugenia et al. found that lithium pretreatment prevented the apoptosis of subgranular cells and preserved neurocognitive function after cranial irradiation in mice [133]. Lithium also reduced mechanical damage to neurons caused by solid stress in brain tumors [134]. Additionally, lithium is effective for treating acute renal injury during cancer therapy. Lithium reduced the inflammation of renal tubules induced by bacterial infection [135,136]. Mechanically, lithium activated Akt and enhanced the reabsorption of the tubule to ease albumin overload [137]. Lithium also reduced renal ROS levels and prevented mitochondrial dysfunction caused by gentamicin [138]. In addition, lithium protected against acute renal injury induced by cisplatin via stimulation of autophagy [138].

Rationally, lithium does not synergize with all antitumor drugs. For example, when used with etoposide or camptothecine, lithium appeared to have an antagonistic effect [139]. Moreover, by inducing autophagy, lithium inhibited apoptosis of neuroblastoma by imatinib mesylate or rotenone treatment [139,140].

## 5. Clinical Applications of Lithium Nowadays

### 5.1. Anti-Leukopenia in Chemotherapy

Lithium carbonate improves hematopoietic function, which is inhibited by traditional chemotherapy [109]. Commonly, chemotherapy is restricted by the occurrence of thrombocytopenia and leukopenia. The period of leukopenia is reduced by lithium carbonate treatment with significantly increasing neutrophils [18]. The treatment also slightly increases the level of eosinophils and basophils [117]. Currently, lithium carbonate is used in clinical leukopenia treatment as the chemotherapy adjuvant. Moreover, another clinical study found that lithium carbonate restored platelets by releasing colony-stimulating factors and IL-6, reaffirming its effect on hematology [118].

### 5.2. Adjuvant of ^131^I for Ablation Therapy

Radioactive iodine (^131^I) is used for thyroid remnant ablation during thyroidectomy to prevent relapse [19]. However, about 20% of the patients with thyroid tumor still have a risk of recurrence after ^131^I treatment [141]. Lithium carbonate significantly increases iodine retention in differentiated thyroid carcinoma, and thyroid remnant reduces thyroid function and fixes the ablation therapy success rate to nearly 100% [19].

## 6. Clinical Challenges Associated with Lithium

### 6.1. Lithium Has Biphasic Effects on Tumor Cell Proliferation and Apoptosis

Lithium exhibits opposite effects on cell proliferation and apoptosis at different concentrations for different cancer types (Table 2). In most conditions, a low concentration of lithium (≤5 mM) inhibited p53 and Bax expression, increasing p21 and survivin levels, and prevented DNA fragmentation [142,143], while a high concentration of lithium (≥50 mM) induced apoptosis and inhibited the DNA replication [143]. It was also found that midkine, a heparin-binding, anti-apoptotic growth factor, increased after a low concentration (1–10 μM) of lithium treatment, but down-regulated after lithium concentration reached 100 μM [63]. Therefore, a low level of lithium may benefit tumor development, while a high level suppresses tumor growth.

**Figure 3:**
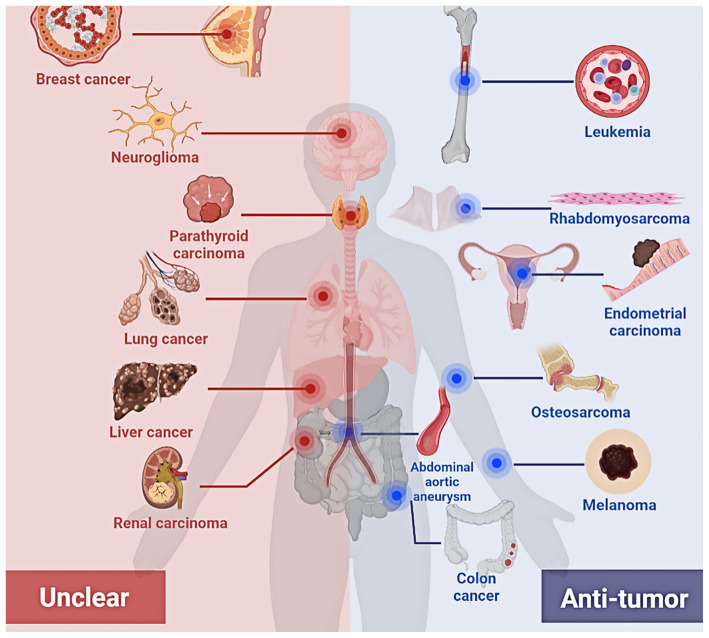
Effects of lithium on tumors of different organ origins. Studies at the cellular and animal levels have shown that lithium exerts antitumor effects in some tumors of specific organ origin (shown in the blue zone), but the effects on others remain elusive (shown in the red zone).

Lithium exhibits its biphasic efficiency for cancer treatment in different cancers. For example, 2 mM lithium stimulated p21 and survivin expression, and promoted malignant glioma development [144]. Differently, 0.1 mM lithium stimulated apoptosis in glioblastoma multiform cells [147]. These results suggest that more pre-clinical and clinical research is required for lithium to be applied to cancer therapy (Figure 3). Different mechanisms may exist in different types of tumors after lithium treatment.

### 6.2. The Narrow Therapeutic Window and High Dosage Requirement of Lithium

Anthony et al. have summarized the challenge of lithium in BD therapy in 2021 [5]. Briefly, the application of lithium is hindered by its narrow therapeutic window, poor patient compliance, and potential toxic injury with chronic treatment (over 1 year). For clinical purposes, lithium levels in plasma remain around 0.6 to 1.0 mEq/L. When lithium concentrations exceed 1.5 mEq/L, mild side effects occur, and lethal effects occur over 2 mEq/L. Normally, a consistent inspection is required to limit lithium levels in serum under 1.2 mEq/L [5,6,148]. Furthermore, the patient’s non-adherence is mainly attributed to the lithium’s unpleasant side-effect profile, which is managed with strategies such as finding antidotes for specific side effects, changing to a different lithium formulation, and altering the time of medication administration [6].

Fortunately, the problem of lithium, which requires a higher concentration in the serum to overcome the blood-brain barrier for psychosis, is not reflected in cancer therapy. However, basing pre-clinical studies on cancer therapy, a higher concentration is acquired for therapeutic efficiency in cancer than in psychosis. Although, the side effects of lithium are mostly mild and non-lethal, its potential toxicity to the renal system and liver might weaken a patient’s tolerance to other first-line drugs for cancer therapy.

### 6.3. The Immune Inhibitory Effects of Lithium

Generally, lithium is considered an anti-inflammation drug to fight against neuro-inflammation [149,150,151]. For example, BD patients expressed lower IL-2, IL-6, IL-10, and IFN-γ in their peripheral blood after a three-month lithium treatment [152]. Lithium also decreased the LPS-induced pro-inflammation cytokines in peripheral blood mononuclear cells via suppressing GSK3β activity [153]. However, this anti-inflammation effect of lithium seems unsuitable for all conditions. In erythematosus patients, higher IL-1β, IL-2, IL-6, IL-17, and TNF-α were observed in whole blood cells stimulated with lithium [154]. Cai et al. found that lithium enhanced the efficiency of IL-2 for the immunotherapy of lymphokine-activated killer cells in a melanoma model [155]. Hence, lithium may have pro-inflammatory effects within an abnormal immune-microenvironment.

The cytokine secretion profile is not sufficient to describe the effect of lithium on the immune system. More research, focused on the development and activation of different types of immune cells, is needed. Lithium was found to induce immature DC to monocyte DC (MoDCs), with up-regulation of DC markers CD86 and CD83 [156], but simultaneously inhibit the differentiation of mature MoDCs and convert them to macrophage-like cells [157]. Furthermore, lithium tends to induce mature MoDCs to release more IL-10, and IL-5 and transfer CD4^+^ T cells to the pro-tumor Th2 subtype [158]. Additionally, lithium modulated the polarization of macrophages to prevent the antitumor M1 polarization and promote the pro-tumor M2 polarization with a higher IFN-γ and lower IL-10 [159,160]. We believe that more studies should be conducted to explore the role of lithium in tumor immunotherapy.

## 7. Conclusions and Prospects

**Figure 4:**
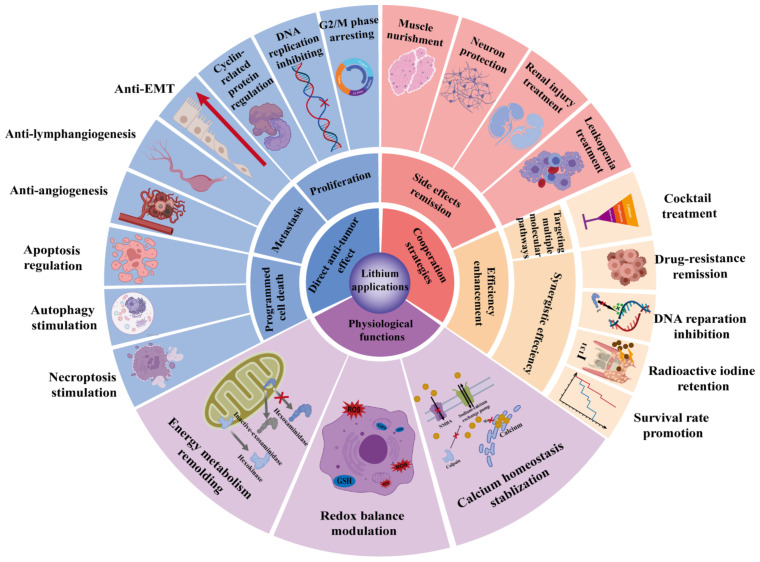
The landscape of lithium in cancer. The tumor-biological roles of lithium provide new sights into its potential for cancer therapy. Lithium inhibits tumor development mainly by suppressing proliferation and metastasis, and promoting programmed cell death. Lithium may reduce side effects and enhance the efficiency of several traditional therapies against cancer.

In summary, as a first-line drug that has been used clinically in BD treatment, the role of lithium in cancer treatment has been gradually gaining attention in recent years (Figure 4). Laboratory studies based on cell lines and animal models have demonstrated the antitumor effects of lithium in several specific cancer types, including colon cancer, endothelial cancer, melanoma, osteosarcoma and leukemia. Lithium exerts antitumor effects through an inhibition of proliferation and migration, or induction of programmed cell death, such as apoptosis. Although the exact molecular mechanism remains to be further explored, it is generally believed that the antitumor effect of lithium is related to its inhibition of GSK3β or IMPase. The development and application of technologies, such as single-cell RNA sequencing, will help reveal the antitumor mechanism of lithium more comprehensively in the future.

Although the safety of lithium in BD therapy has been extensively described, the blurred line between the high dose required for lithium to exert its antitumor effects and its toxic dose is one of the major challenges currently limiting lithium antitumor research. The limited clinical studies available suggest that lithium may increase the therapeutic efficacy and reduce the side effects of some anti-cancer drugs. Combination therapy remains an important future direction for the exploration of the antitumor effects of lithium. Considering the nature of lithium as a metal drug, we believe that the narrow therapeutic window problem may be solved by the construction of some controlled-releasing delivery systems. In addition, most of the reported effects of lithium on the immune system have been established in non-tumor disease models. The potential role of lithium in the tumor microenvironment deserves further explorations.

## Figures and Tables

**Table 1:** Clinical studies of lithium in cancer therapy.

Ref. (Year)	Cancer Type	Lithium Salts	Dosage	Sample Size (N)	Cooperated Therapy	Comments of Efficacy
[116] (2001)	Pancreatic cancer	LiGLA	700 mg once a day	278	N/A	Lithium lacked demonstrable efficacy.
[117] (2002)	Ewing sarcoma/rhabdomyosarcoma/pancreatic cancer	Li_2_CO_3_	300 mg three times daily	100	Combined with daunorubicin, cytosine arabinoside, and thioguanine and radiotherapy	Lithium increased the number of neutrophil granulocytes significantly. Lithium increased the number of eosinophil granulocytes and lymphocytes significantly
[118] (2001)	Ependymoblastoma/fibrosarcoma/malignant/teratoma/neuroblastoma	Li_2_CO_3_	300 mg three times daily	100	Combined with daunorubicin, cytosine arabinoside, and thioguanine and radiotherapy	Statistically and clinically significant leukocyte proliferation could be observed when using lithium for immunotherapy. A significant increase in the mean number of platelets for patients was observed after lithium treatment.
[112] (1996)	Pancreatic cancer	LiGLA	7 g/77 g once a day	48	N/A	The highest doses of lithium were associated with longer survival times as compared with the lowest doses.
[119] (2004)	Hepatocellular carcinoma/giant-cell tumor of the bone/renal cell carcinoma	LiGLA	750 mg once a day	45	Conjugated with iodized lymphographic oil	A significant reduction in the size of the tumor was observed.
[111] (1981)	Small-cell lung cancer	Li_2_CO_3_	300 mg three times daily	45	Combined with daunorubicin, cytosine arabinoside, and thioguanine	Lithium demonstrated a higher objective response rate and longer survival.
[120] (1984)	AML	Li_2_CO_3_	300 mg three times daily	41	Combined with cytosine arabinoside and daunorubicin	The duration of neutropenia was not significantly shorter for patients receiving lithium than for controls.
[20] (1980)	Ewing sarcoma/rhabdomyosarcoma/nasopharyngeal carcinoma/osteogenic sarcoma/ependymoblastoma/fibrosarcoma/malignant/teratoma/neuroblastoma	Li_2_CO_3_	150 mg and 300 mg once a day	39	Combined with daunorubicin, cytosine arabinoside, and thioguanine and radiotherapy	Lithium reduced the period of leukopenia after chemotherapy. During lithium treatment, patients might suffer infections.
[19] (2012)	Differentiated thyroid carcinoma	Li_2_CO_3_	300 mg three times daily	29	Near-total thyroidectomy was accepted before the study; ^131^I therapy	Lithium treatment improved the efficacy of thyroid remnant ablation.
[121] (1989)	AML	Li_2_CO_3_	1200 mg Once a day	29	combined with daunorubicin, cytosine arabinoside, and thioguanine	The number of remissions, relapse-free survival, and survival were similar for the lithium-treated and control groups. There was no apparent clinical efficacy in the use of lithium to reduce the period of neutropenia in patients undergoing remission induction therapy for acute myeloid leukemia.
[122] (1979)	AML	Li_2_CO_3_	300 mg three times daily	27	Combined with cytosine arabinoside and thioguanine	Lithium-treated group showed a shortened duration of neutropenia. The incidence of infections and the rate of remission were not affected by lithium.
[123] (1988)	AML	Li_2_CO_3_	250 mg three times daily	26	N/A	No difference was observed in complete remission rates and disease-free survival between the two groups. A significant reduction in the number of days of antibiotic therapy required was found in the treated group.
[124] (1980)	Colorectum cancer/stomach cancer/breast cancer/lung cancer/ovary cancer/tongue cancer/astrocytoma/plasmocytoma/Hodgkin’s disease/pancreas cancer/esophagus cancer/larynx cancers	Li_2_CO_3_	250 mg three times daily	26	Combined with 5-ftuorouracil, lomustine	Lithium was capable of raising the leukocyte count to a highly significant extent, without serious side effects. The leukocytosis was due to an increase in neutrophil granulocytes.
[18] (1980)	Small-cell bronchogenic carcinoma	Li_2_CO_3_	300 mg three times daily	23	Combined with cyclophosphamide, doxorubicin, and vincristine	Infection-free survival was significantly longer in the lithium-treated group. The lithium-treated group suffered fewer neutropenia days.
[125] (1984)	Small-cell lung cancer	Li_2_CO_3_	300 mg three times daily	20	Combined with doxorubicin and vincristine	Lithium administration was associated with a greater risk of sudden death and shorter survival. Lithium carbonate significantly reduced infection risk, but increased cardiomyopathy risk cooperating with anthracycline antibiotics.
[126] (2021)	Breast cancer	Li_2_CO_3_	300 mg three times daily	18	N/A	Lithium was not advantageous or beneficial for the prevention of CIPN. Electromyography and nerve conduction velocity variables became better in the lithium group without significance.
[127] (2011)	Low-grade neuroendocrine tumors	LiCl	300 mg three times daily	15	N/A	Lithium lacked demonstrable efficacy.
[128] (1999)	Differentiated thyroid carcinoma	Li_2_CO_3_	300 mg three times daily	15	Near-total thyroidectomy was accepted before the study; ^131^I therapy accepted	More ^131^I accumulated during lithium therapy.
[110] (2020)	AML	Li_2_CO_3_	300 mg three times daily	9	N/A	Lithium induced differentiation of AML stem cells.
[129] (2017)	Glioblastoma	Li_2_CO_3_	400 mg three times daily	7	Combined with cimetidine, olanzapine, and valproate	Lithium showed significantly longer survival.
[130] (2022)	Breast cancer	N/A	N/A	914	N/A	All antipsychotic drugs (including lithium) are associated with a 35% increased risk of breast cancer.
[131] (2019)	Breast cancer	N/A	N/A	326	N/A	All psychotropic medication use was not associated with invasive breast cancer risk.

**Table 2:** The bipolar effects of lithium on tumor cells.

Refs.	Cancer Type	Dosage	Effects	Direction
[143]	Breast cancer	50–100 mM	Lithium reduced cell viability and down-regulated the ratio of Bcl-2/Bax.	Antitumor
[40]	Colon cancer	10–60 mM	Lithium promoted ROS production, down-regulated cell viability, and lowered the expression of Bcl-2 and survivin.	Antitumor
[144]	Glioblastoma	20 mM	Lithium reduced cell viability.	Antitumor
[73]	Leukemia	12.5–30 mM	Lithium inhibited cell viability and induced apoptosis.	Antitumor
[46]	Lung cancer	20 mM	Lithium stimulated the overexpression of DR4 and DR5 and enhanced the efficiency of TRAIL.	Antitumor
[95]	melanoma	5–20 mM	Lithium separated hexokinase from mitochondria in a dosage manner.	Antitumor
[140]	Neuroblastoma	50 mM	Lithium reduced cell viability.	Antitumor
[68]	Osteosarcoma	20 mM	Lithium inhibited DNA replication and cell migration.	Antitumor
[63]	Prostate cancer	100–500 μM	Lithium down-regulated MK level, reduced cell viability, and induced apoptosis.	Antitumor
[51]	Rhabdomyosarcoma	10–25 mM	Lithium inhibited cell proliferation and enhanced the efficiency of arsenic trioxide.	Antitumor
[143]	Breast cancer	1–10 mM	Lithium up-regulated the ratio of Bcl-2/Bax and improved cell viability.	Pro-tumor
[142]	Breast cancer	110 mM	Lithium up-regulated Bcl-2, down-regulated p53 level, and then improved cell viability	Pro-tumor
[144]	Glioblastoma	0.5/2/20 mM	Lithium up-regulated p21 and survivin levels in a dosage-dependent manner.	Pro-tumor
[139]	Hepatoblastoma/ lung carcinoma/breast carcinoma	20 mM	Lithium down-regulated CD95 via inactivation of p53 and then suppressed apoptosis induced by etoposide and camptothecin.	Pro-tumor
[145]	Neuroblastoma/ Neuron	0.5–2 mM	Lithium down-regulated cytochrome C, up-regulated the ratio of Bcl-2/Bax and prevented mitochondrial damage.	Pro-tumor
[91]	Neuroblastoma/ Neuron	0.5/0.7 mM	Lithium increased cell viability significantly and down-regulated ROS levels.	Pro-tumor
[140]	Neuroblastoma	1–50 mM	Lithium prevented rotenone-induced apoptosis and mitochondrial dysfunction.	Pro-tumor
[63]	Prostate cancer	1–10 μM	Lithium up-regulated cell viability and MK level.	Pro-tumor
[146]	Retinoblastoma	20/40 mM	Lithium enhanced DNA replication and increased the portion of stem cells.	Pro-tumor

## Data Availability

Not applicable.
